# Recombinant antithrombin attenuates acute kidney injury associated with rhabdomyolysis: an in vivo animal study

**DOI:** 10.1186/s40635-024-00594-y

**Published:** 2024-01-29

**Authors:** Tomotaka Miura, Tomoki Okuda, Kodai Suzuki, Hideshi Okada, Hiroyuki Tomita, Chihiro Takada, Kosuke Mori, Hirotaka Asano, Soichiro Kano, Yugo Wakayama, Yohei Fukuda, Hirotsugu Fukuda, Ayane Nishio, Yuki Kawasaki, Ayumi Kuroda, Keiko Suzuki, Ryo Kamidani, Haruka Okamoto, Tetsuya Fukuta, Yuichiro Kitagawa, Takahito Miyake, Keita Nakane, Akio Suzuki, Takahiro Yoshida, Nobuyuki Tetsuka, Shozo Yoshida, Takuya Koie, Shinji Ogura

**Affiliations:** 1 Department of Emergency and Disaster Medicine, Gifu University Graduate School of Medicine, 1-1 Yanagido, Gifu, 501-1194 Japan; 2 Department of Infection Control, Gifu University Graduate School of Medicine, Gifu, Japan; 3 Center for One Medicine Innovative Translational Research, Gifu University Institute for Advanced Study, Gifu, Japan; 4 Department of Tumor Pathology, Gifu University Graduate School of Medicine, 1-1 Yanagido, Gifu, 501-1194 Japan; 5 Department of Pharmacy, Gifu University Hospital, Gifu, Japan; 6 Abuse Prevention Center, Gifu University Graduate School of Medicine, Gifu, Japan; 7 Department of Urology, Gifu University Graduate School of Medicine, Gifu, Japan

**Keywords:** Acute kidney injury, Rhabdomyolysis, Recombinant antithrombin, In vivo mice model, Novel treatment

## Abstract

**Background:**

Rhabdomyolysis is characterized by the destruction and necrosis of skeletal muscle tissue, resulting in acute kidney injury (AKI). Recombinant antithrombin (rAT) has DNA repair and vascular endothelial-protection properties. Herein, we investigated whether rAT therapy has beneficial effects against rhabdomyolysis-induced AKI. Ten-week-old male B6 mice were injected with 5 mL/kg of 50% glycerol intramuscularly in the left thigh after 24 h of fasting to create a rhabdomyolysis mouse model. Further, 750 IU/kg rAT was injected intraperitoneally at 24 and 72 h after the rhabdomyolysis model was established. The mice were euthanized after 96 h for histological analysis. Saline was administered to mice in the control group.

**Results:**

Blood tests show elevated serum creatinine, urea nitrogen, and neutrophil gelatinase-associated lipocalin levels in rhabdomyolysis. Loss of tubular epithelial cell nuclei and destruction of the tubular luminal surface structure was observed in the untreated group, which improved with rAT treatment. Immunostaining for Ki-67 showed increased Ki-67-positive nuclei in the tubular epithelial cells in the rAT group, suggesting that rAT may promote tubular epithelial cell regeneration. The microvilli of the brush border of the renal tubules were shed during rhabdomyolysis, and rAT treatment reduced this injury. The vascular endothelial glycocalyx, which is usually impaired by rhabdomyolysis, became functional following rAT treatment.

**Conclusions:**

Treatment with rAT suppressed rhabdomyolysis-induced AKI, suggesting that rAT therapy may be a novel therapeutic approach.

**Supplementary Information:**

The online version contains supplementary material available at 10.1186/s40635-024-00594-y.

## Background

Rhabdomyolysis is a clinical syndrome characterized by damage to skeletal muscle fibers and leakage of myocyte contents into the circulation, including electrolytes and sarcoplasmic reticulum proteins, such as myoglobin, creatinine kinase, aldolase, lactate dehydrogenase, and aspartate aminotransferase [1].

Damaged skeletal muscles are broken, resulting in the release of contents into the bloodstream, which is filtered by glomeruli, causing tubular obstruction and inflammation due to protein precipitation [2]. Particularly, the release of myoglobin induces severe oxidative damage because the heme portion induces lipid peroxidation and renal tubular injury, which is one of the main factors in the development of acute kidney injury (AKI) [3]. Hence, rhabdomyolysis-induced AKI is predominantly a renal tubular injury rather than a glomerular injury.

Rhabdomyolysis is one of the most common causes of AKI, accounting for up to 50% of all AKI cases, with a mortality rate as high as 5–10% [4, 5]. Furthermore, AKI has been associated with intrarenal and systemic inflammation [6]. However, there is no curative treatment for AKI except symptomatic therapy, such as transfusion and blood purification.

Antithrombin III (AT) has inhibitory activity against thrombin and acts as an anticoagulant by inhibiting the interaction between thrombin and activated coagulation factors [7]. In addition, AT binds to heparin sulfate on endothelial cells and syndecan-4 on neutrophils and has been reported to inhibit inflammation activated by lipopolysaccharide and endothelial glycocalyx damage through tissue repair actions, indicating its protective role against systemic inflammation [8–10].

Therefore, we hypothesize that AT tissue repair, including vascular endothelial cells, would have a beneficial effect on AKI caused by rhabdomyolysis. This study aimed to determine whether recombinant AT (rAT) therapy has beneficial effects against rhabdomyolysis-induced AKI.

## Methods

### In vivo animal study

This study was approved by the Institutional Animal Research Committee of Gifu University (2022-053, Gifu, Japan). Ten-week-old male C57BL6 mice (RRID: MGI: 5657202; Charles River Laboratories, Japan, Inc., Yokohama, Japan) were housed in a colony room maintained at 25 °C and a 12:12 h light/dark cycle. The mice had free access to powdered mouse chow (CE-2 Rodent Diet, Nihon CLEA Ltd., Tokyo, Japan) and tap water.

To induce rhabdomyolysis, 5 mL/kg of 50% glycerol was injected intramuscularly into the left thigh of the mice, and the same volume of saline was injected intramuscularly into the sham-operated mice, as previously described [11]. The animals fasted with water for 24 h before and after the injection of glycerol and saline solutions. The mice were intraperitoneally administered rAT (750 IU/kg; Kyowa Kirin Co., Ltd., Tokyo, Japan) 48 and 72 h after glycerol injection. Saline was administered to the control group in the same manner. The mice that survived were euthanized 96 h after glycerol injection and kidney specimens were collected. Perfusion fixation and blood sampling from the buccal artery were performed under anesthesia (using a mixture of medetomidine hydrochloride 0.3 mg/kg, midazolam 4 mg/kg, and butorphanol tartrate 5 mg/kg, given intraperitoneally). The anesthesia was deemed sufficient if the corneal and hind-paw withdrawal reflexes were absent. Before kidney specimens were obtained, the mice were killed by exsanguination from the buccal artery until the righting reflex was lost.

### Serum preparation and blood examination

Blood samples were collected from six mice from the buccal artery. Blood was allowed to clot at 25 °C for 2 h and centrifuged (2000×*g* at 4 °C for 20 min). Thereafter, the supernatant was collected. Serum blood urea nitrogen and creatinine levels were measured using SRL (Hachioji, Tokyo, Japan). Serum neutrophil gelatinase-associated lipocalin (N-GAL) levels were measured using enzyme-linked immunosorbent assay quantitation kits for mice (KIT042; BioPorto Diagnostics Inc., Hellerup, Denmark).

### Histopathological assessment

The whole right kidneys of six mice, 96 h after glycerol administration, were fixed in phosphate-buffered saline (PBS) containing 10% formalin and then embedded in paraffin. The paraffin sections (4-µm-thick) were then de-paraffinized and rehydrated. Finally, the slides were counterstained with hematoxylin and eosin. Then, to quantitative assess the percentage of tubules in the outer medulla that showed epithelial cell necrosis, a numerical score was assigned to represent the degree of injury as follows: 0, no injury; 1, 0–10%; 2, 11–20%; 3, 21–40%; 4, 41–60%; 5, 61–75%; and 6, > 75% [12]. These experiments were performed in a blinded manner to avoid bias.

### Immunohistochemistry and image analysis

The de-paraffinized 4-μm-thick sections were incubated with primary antibodies against Ki-67 (ab16667; Abcam, Cambridge, UK), proliferating cell nuclear antigen (PCNA; M0879; DAKO, Santa Clara, CA, USA), and the endothelial cell marker CD31 (M0823; DAKO, Santa Clara, CA, USA). The localization of the target proteins was visualized using the VECTASTAIN Elite ABC system (Vector Laboratories, Newark, CA, USA) or secondary antibodies (Alexa Fluor 488 and 568; Invitrogen, Carlsbad, CA, USA). The nuclei were stained with hematoxylin or Hoechst stain. The number of Ki67-positive and PCNA-positive cells was counted to quantify the number of cells in which DNA replication occurred. Cell counts were performed in 10 randomly selected high-magnification fields of view (HPF) in each section (*n* = 6).

For quantitative analysis of the fluorescence intensity of the endothelial glycocalyx, the line scan profile analysis of the vessel wall was selected as a representative of red fluorescent tomato-lectin accumulation, and the vessel boundary was confirmed by green, fluorescent CD31 expression. The intensity profiles of two channels, red and green, were measured using the Image J software (National Institutes of Health, Bethesda, MD, USA). Ten images per mouse were selected from six mice in each group, and three lines were calculated randomly in each image.

### Electron microscopy

Electron microscopic analysis of the renal tubules was performed as previously described [13]. Briefly, for the visualization of the endothelial glycocalyx, the mice were anesthetized and then perfused with a solution comprising 2% glutaraldehyde, 2% sucrose, 0.1 M sodium cacodylate buffer (pH 7.3), and 2% lanthanum nitrate, at a steady flow rate of 1 ml/min, through a cannula placed in the left ventricle. After sacrificing the mice, the kidney samples were fixed in a solution without glutaraldehyde and washed in an alkaline (0.03 M NaOH) 2% sucrose solution. The freeze-fracture method was used to prepare the samples for scanning electron microscopy (SEM; S-4800, Hitachi, Tokyo, Japan). To prepare samples for conventional electron microscopy, 2.5% glutaraldehyde in 0.1 M phosphate buffer (pH 7.4) without lanthanum nitrate was used as the fixative.

To prepare samples for transmission electron microscopy (TEM), each specimen was embedded in epoxy resin. Ultrathin sections (90 nm), stained with uranyl acetate and lead citrate, were then examined using TEM (HT-7700, Hitachi). For sample fixation, 2.5% glutaraldehyde in 0.1 mol/l phosphate buffer (pH 7.4) without lanthanum nitrate was used.

Electron microscopy was performed on six independent kidney samples from individual mice in each group.

### Statistical analyses

Data are presented as the mean ± standard error. The two groups with six samples per group were compared using the Mann–Whitney *U* test. *P* < 0.05 was considered statistically significant. All calculations were performed using the GraphPad Prism software (La Jolla, CA, USA). To determine the number of cases in this study, power calculation was conducted, aiming at a power of 80%, a significance level of 5%, and an effect size for histological scoring of 1.66. The effect size was the difference between the value of the histological scoring variable in the saline group and that in the treatment group determined using EZR (Saitama Medical Center, Jichi Medical University, Saitama, Japan; https://www.jichi.ac.jp/saitama-sct/SaitamaHP.files/statmedEN.html), a graphical user interface for R version 4.2.2 (The R Foundation for Statistical Computing, Vienna, Austria).

## Results

### The morphological differences in the capillaries between the glomerulus and renal tubules in the normal state

**Fig. 1:**
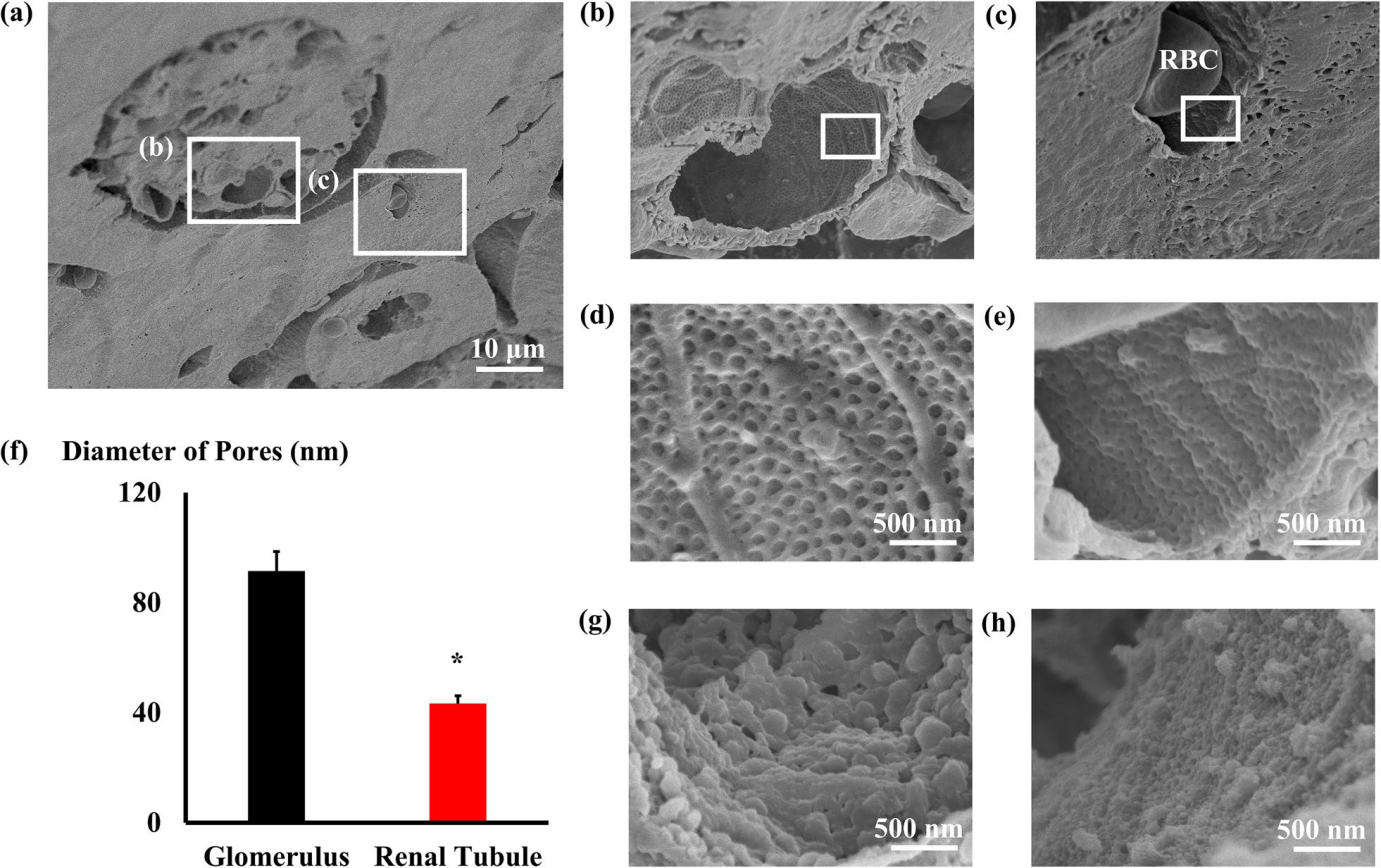
Scanning electron microscope images of the glomerular or renal tubular endothelial cells. **a** Representative image of a sham-operated mouse kidney. **b** Glomerular capillary (the expanded image of the white square in **a**. **c** Renal tubular capillary (the expanded image of the white square in **a**. **d** Surface of a glomerular endothelial cell in the white square in **b**. **e** Surface of a renal tubular endothelial cell of the white square in **c**. **f** Graphs of the diameters of the pores in glomerular and renal tubular endothelial cells *P < 0.01 vs. diameters of the pores in glomerular endothelial cells (*n* = 6 each group). **g**, **h** Representative endothelial glycocalyx images were visualized using lanthanum nitrate in the glomerular capillary (**g**) and renal tubular capillary (**h**)

The shape of the glomerular capillaries was classified as fenestrated. In the sham-operated mice, the glomerular and renal tubular capillaries had many small pores in the endothelial cells (Fig. 1a–e). However, the pore size of the renal tubules was significantly smaller than that of the glomerular capillaries (Fig. 1f). In addition, the shapes of the endothelial glycocalyces observed with lanthanum nitrate were significantly different between the two capillaries (Fig. 1g, andh). The endothelial cell shape could be observed in the renal tubular capillaries due to the thin glycocalyx on the surface of the capillaries (Fig. 1h); however, in the glomerular capillaries, the glycocalyx was thick, and the endothelial cell shape could not be observed (Fig. 1g).

### rAT attenuated AKI due to glycerol-induced rhabdomyolysis

**Fig. 2:**
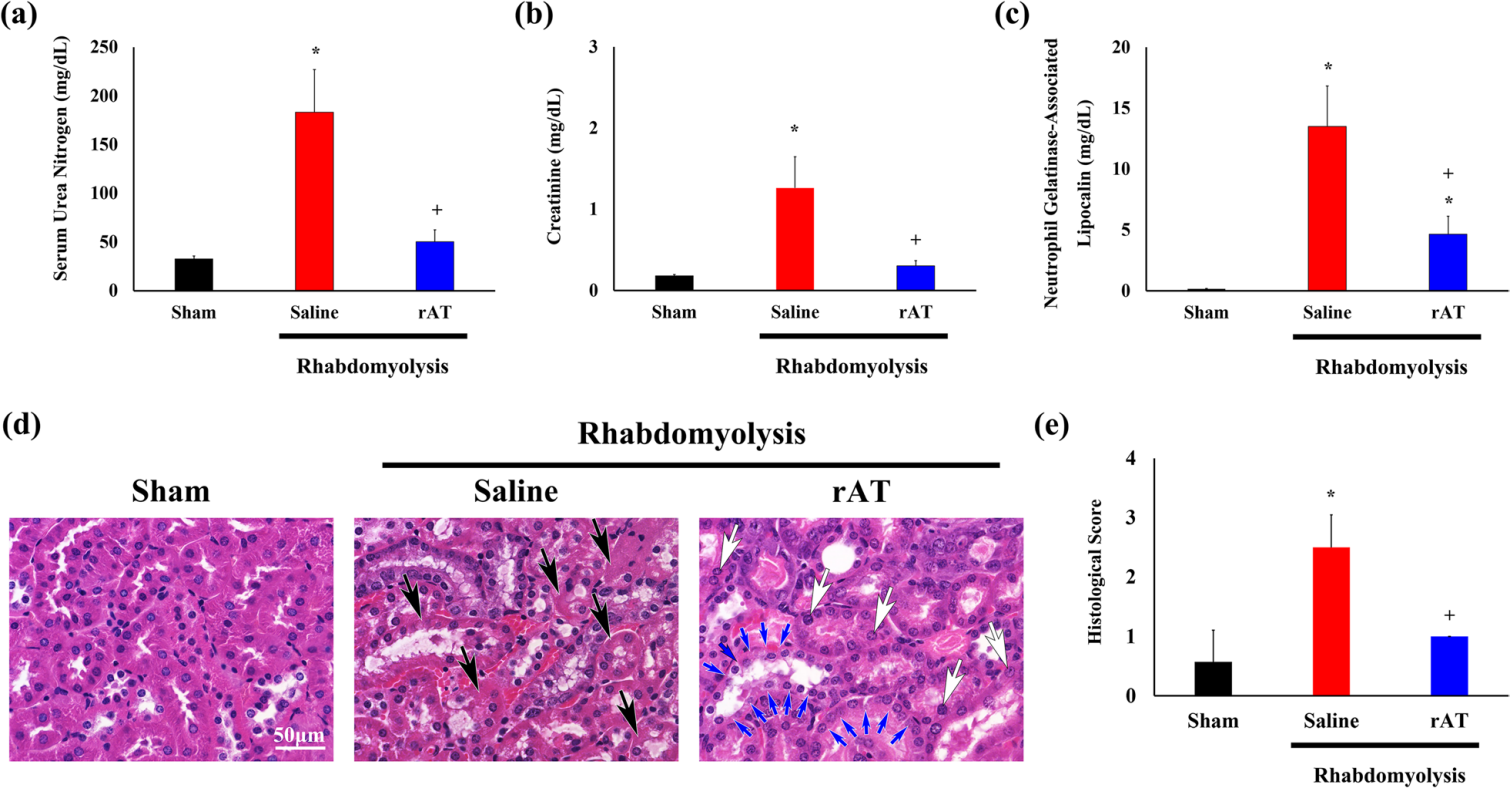
Recombinant antithrombin (rAT) treatment ameliorated acute kidney injury induced by rhabdomyolysis. **a**–**c** Serum blood urea nitrogen (**a**), creatinine (**b**), and neutrophil gelatinase-associated lipocalin (**c**) concentrations in mice measured using an enzyme-linked immunosorbent assay (*n* = 6 in each group). **d** Hematoxylin and eosin-stained renal tissues, with arrows indicating the loss of tubular epithelial cell nuclei (black arrows), enlarged tubular epithelial cell nuclei (white arrows), and tubular meandering (blue arrows). **e** Graph showing the histological scores of renal injury (*n* = 6 in each group). *P < 0.05 vs. sham-operated mice; ^+^*P* < 0.05 vs. saline-injected mice. Scale bars: 50 µm (**d**)

Serum blood urea nitrogen, creatinine, and N-GAL levels were significantly elevated 96 h after glycerol administration in the saline-treated group compared to those in the sham-operated mice (Fig. 2a–c). However, this elevation was attenuated in the rAT-injected mice compared to the saline-treated mice. These results indicated that glycerol-induced rhabdomyolysis caused AKI and that rAT treatment attenuated this effect.

### rAT treatment suppressed renal tubular injury due to glycerol-induced rhabdomyolysis

Under rhabdomyolysis conditions, many nuclei of the tubular epithelial cells were lost in saline-treated mice (Fig. 2d), and the nuclei were unevenly aligned. Further, the lumen of the tubules was enlarged, and the brush border was absent, indicating tubular necrosis. In contrast, some tubular epithelial cells were nucleated and enlarged (Fig. 2D). There was also evidence of tubular meandering, suggesting that the tubular epithelium regenerated in the rAT-treated mice (Fig. 2d). Histological scores indicated that the percentage of tubules in the outer medulla of the necrotized epithelial cell worsened in saline-treated mice and improved in rAT-treated mice (Fig. 2e).

### rAT may be associated with renal tubular regeneration

**Fig. 3:**
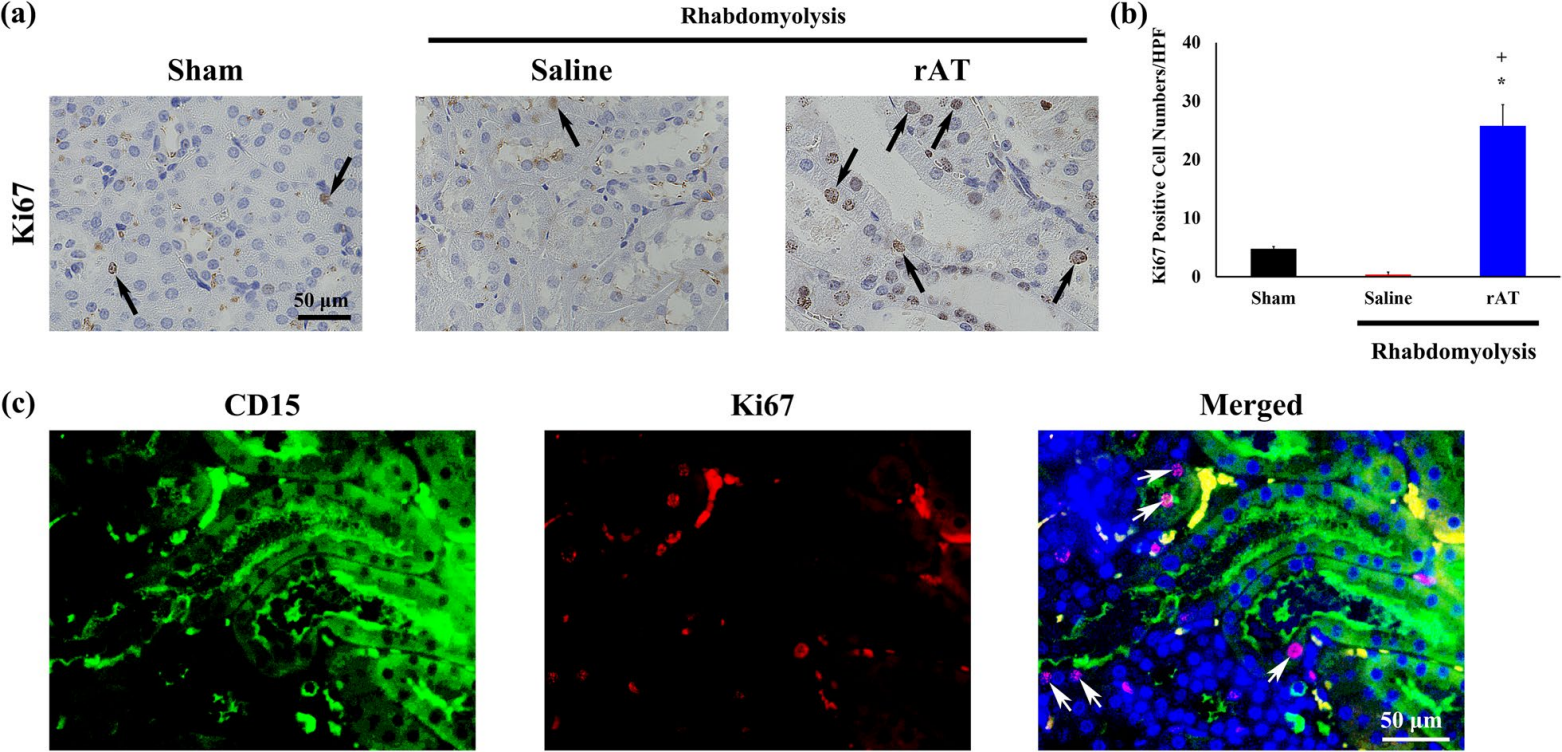
Immunohistochemical analysis of Ki-67. **a** Representative images of the immunohistochemical staining for Ki-67 in the kidneys of sham-operated mice and mice treated with saline or rAT. Black arrows indicate Ki-67-positive cells. **b** Graph showing the number of Ki-67-positive cells (*n* = 6 in each group). **c** Double immunofluorescence staining for Ki-67 and CD15. White arrows indicate colocalization of Ki-67 and CD15 expression in proximal renal tubular cells. *P < 0.05 vs. Sham-operated mice. ^+^*P* < 0.05 vs. Saline-treated mice. Scale bars: 50 µm (**b**, **c**). HPF, high-power field

To examine whether the tubular epithelial cells were proliferating, the tubules were immunohistochemically stained for Ki-67, a marker of cell division (Fig. 3a). The number of Ki-67-positive cells was higher in the kidneys of rAT-treated mice than in those of saline-injected mice (Fig. 3b). To determine cell proliferation, double immunostaining for Ki-67 and CD15, which are markers of renal tubular epithelial cells, was performed, which revealed the colocalization of Ki-67 and CD15 expression in rAT-treated mice (Fig. 3c).

Similarly, the tubules were immunohistochemically stained for PCNA, a factor that promotes DNA polymerase gamma activity (Additional file 1a). The number of PCNA-positive cells in the rAT-treated group was significantly higher than in the saline-treated group, suggesting increased tubular epithelial cell proliferation (Additional file 1b).

### Ultrastructural analysis of the renal tubules

**Fig. 4:**
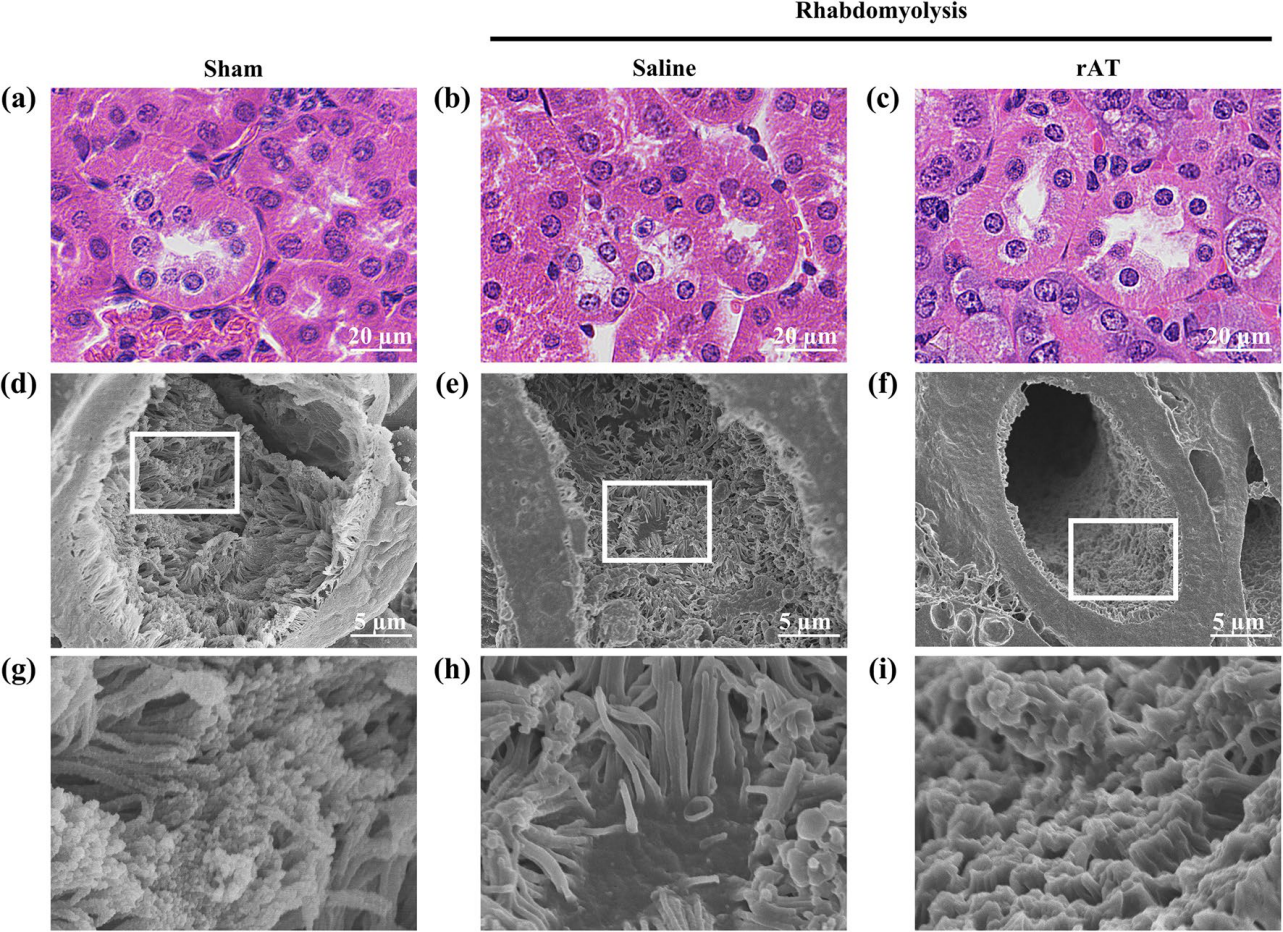
Recombinant antithrombin (rAT) treatment attenuates proximal tubular injury. **a**–**c** Representative hematoxylin–eosin staining images of the proximal renal tubules in sham-operated mice (**a**) and mice treated with saline (**b**) or rAT (**c**). **d**–**f** Representative images of the brush border using a scanning electron microscope for the sham-operated mice (**d**) and mice treated with saline (**e**) and rAT (**f**). **g**–**i** Magnified images of the boxed areas in **d**–**f**, respectively

The brush border is a region of densely formed microvilli of unequal length and thickness in the proximal tubular cells (Fig. 4a–c). SEM was used to observe the ultrastructure of this region.

The proximal tubular lumen of the sham-operated mice was covered with microvilli (Fig. 4d, g and Additional file 2a); however, when the renal injury occurred due to rhabdomyolysis, the density of the microvilli was reduced due to shedding, and some tubular cells were exposed in the lumen (Figure e, h, and Additional file 2b). In contrast, treatment with rAT showed no microvilli shedding in the lumen of the tubules, and their surfaces were covered with microvilli, albeit shorter than in the sham-operated mice (Fig. 4f, i and Additional file 2c, d).

### Ultrastructure of the capillaries perfusing the renal tubules in rhabdomyolysis

**Fig. 5:**
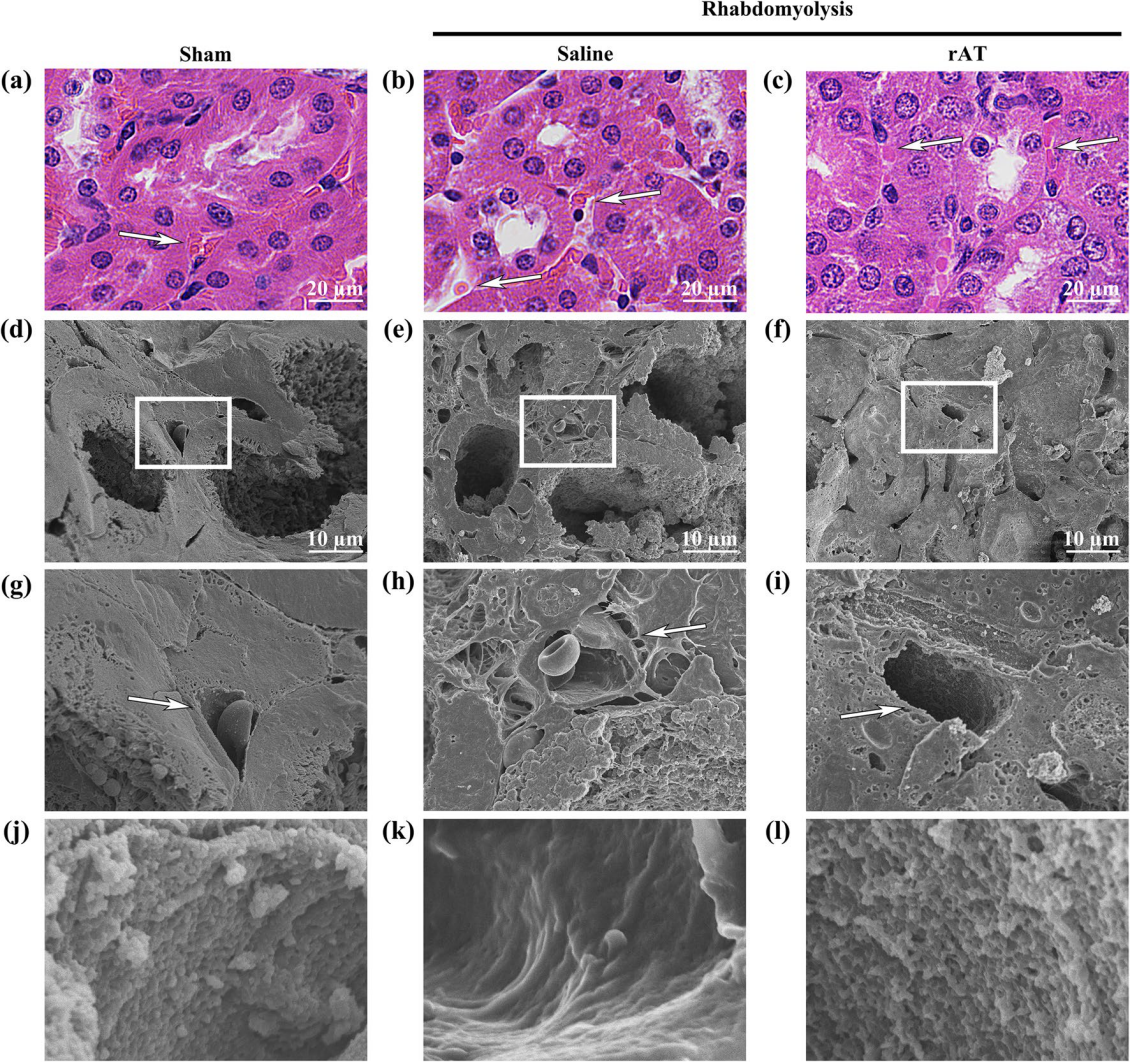
Recombinant antithrombin (rAT) ameliorated rhabdomyolysis-induced endothelial glycocalyx injury. **a**–**c** Representative hematoxylin–eosin staining images of the capillaries that perfused renal tubules in the sham-operated mice (**a**) and mice treated with saline (**b**) and rAT (**c**). White arrows indicate capillaries. **d**–**f** Representative scanning electron microscope images of the capillaries in the sham-operated mice (**d**) and mice treated with saline (**e**) and rAT (**f**). **g**–**i** Magnified images of the white square areas in **d**–**f**, respectively. White arrows indicate the perivascular cavity. **j**–**l** Endothelial glycocalyx images following lanthanum nitrate staining of the center vessels in **g**–**i**, respectively

Capillaries were present in the space between the tubules (Fig. 5a–c), and it was assumed that the tubules were perfused by these vessels. In the sham-operated mice, the tubules and capillaries were in close contact (Fig. 5d and g). However, due to rhabdomyolysis-induced AKI, a gap with fibrosis was created around the capillaries, creating a distance between the capillaries and the tubules (Fig. 5e, andh). In contrast, rAT treatment suppressed perivascular fibrosis; therefore, the capillaries and tubules were closely connected (Fig. 5f and i).

Further examination of the lumen of these vessels revealed that in the sham-operated mice, the lumen was covered with vascular endothelial glycocalyx (Fig. 5j), whereas in the mice with rhabdomyolysis, this structure was shed, exposing the endothelial surface to the vascular lumen and further disrupting the pore structure (Fig. 5k). In contrast, rAT treatment reduced the vascular endothelial glycocalyx injury (Fig. 5l).

### Quantitative assessment of endothelial glycocalyx injury in the renal tubules under rhabdomyolysis condition

**Fig. 6:**
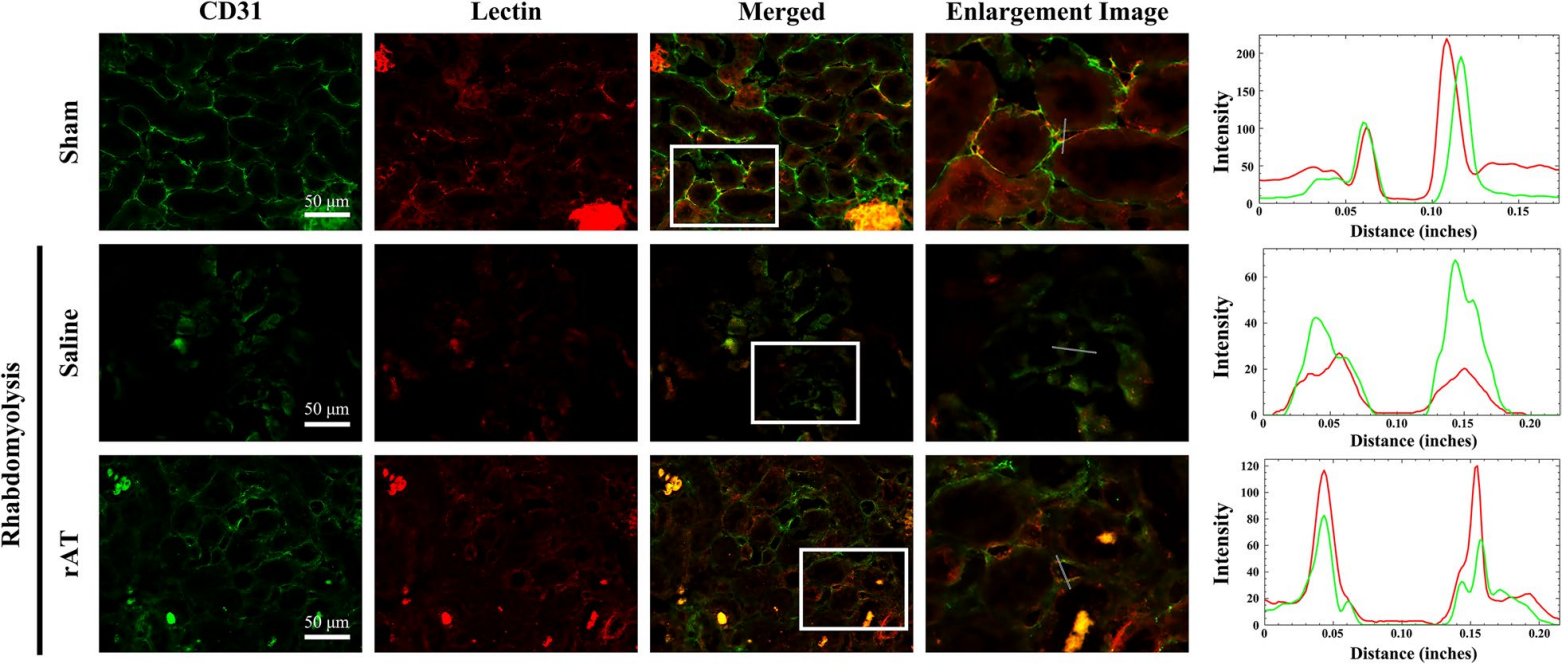
Endothelial glycocalyx injury in the kidney due to rhabdomyolysis was reduced following recombinant antithrombin (rAT) treatment. Representative double-staining images of CD31 immunohistochemistry (green) and wheat germ agglutinin (WGA) lectin (red) in sham-operated mice and mice treated with saline or rAT. The graphs show the line-scan analysis of the white lines of the enlarged images (*n* = 6 in each group)

To examine glycocalyx injury due to the capillary perfusion in the renal tubules, we performed immunohistochemical analysis with CD31 antibody and wheat germ agglutinin (WGA) lectin staining [14], which allowed visualization of the endothelial glycocalyx in the endothelium and helped us assess the staining intensity (Fig. 6). The intensity of WGA lectin staining in CD31-positive areas in the capillaries around the tubules of each group was measured and compared. The intensity was significantly lower in saline-administered mice than in sham-operated mice. However, the intensity was remarkably improved in the rAT-treated group compared to that in the untreated group (Additional file 3).

## Discussion

In this study, we demonstrated that rAT suppresses rhabdomyolysis-induced AKI. The mechanism is discussed, focusing on the relationship between the renal tubules and the vessels that perfuse them.

### Endothelial injury in rhabdomyolysis-induced AKI

The pathogenesis of rhabdomyolysis-induced AKI is associated with the myoglobin released from the muscle [15]. Myoglobin converts to hematin in acidic urine, which is nephrotoxic and causes tubular damage. Myoglobin also constricts renal blood vessels, causing reduced blood flow, which, combined with decreased renal prostaglandin production and vasopressin hypersecretion, is thought to accelerate renal injury due to renal ischemia. Extracellular traps released from macrophages have been reported to be involved in the development of rhabdomyolysis-induced AKI [16]. This present study showed that vascular endothelial damage in AKI occurs due to rhabdomyolysis.

AKI due to sepsis was also previously thought to be acute tubular necrosis due to renal ischemia caused by hypotension and renal vasoconstriction. However, it has been recently suggested that inflammation-related microvascular and tubular functional changes, rather than structural changes, are the main causes of AKI. More recently, functional changes in microvessels and inflammation in tubules, rather than structural changes, have been considered the primary causes [17]. Microvascular damage related to the inflammation identified in this study may cause rhabdomyolysis-induced AKI.

The capillaries extending from the glomerulus to the collecting ducts perfuse the tubules and also pass the primary urine for reabsorption, secretion, and other processes. As shown in our electron microscopy image (Fig. 5g), in normal tissues, tubules and capillaries exist in close proximity and have a morphology suitable for the exchange of substances. However, in rhabdomyolysis-induced AKI, this structure is usually disrupted, and fibrosis develops around the capillaries, creating a void between them and the tubules. Possibly, this morphological change impairs the perfusion of proximal tubular epithelial cells and disrupts the brush border structure of the tubular lumen. In addition, in acute tubular necrosis, necrosis of the proximal tubular epithelium and loss of the brush border have been reported as significant histological differences between the acute and recovery phases [18]. The histological changes observed in this study are consistent with those in previous studies.

### The morphology of capillaries depends on their function

Renal glomerular capillaries are fenestrated with numerous small pore structures in the endothelial cells [13]. In the present study, the vessels perfusing the renal tubules also showed fenestrated morphology, but their structure was somewhat different from that of the glomerulus; that is, the size of the pores and the shape of the glycocalyx on the vascular endothelial cells were different. From a functional point of view, the small pores in glomerular capillaries are thought to be structures for filtering water and substances out of the vessel, whereas those in capillaries perfusing the tubules are thought to be small pores for reabsorbing substances from the tubules. Therefore, the morphological differences in endothelial cells and glycocalyx may be attributed to these functions.

Previous reports have shown that compared with organs with thinner glycocalyx vessels, organs with thicker glycocalyces are less susceptible to injury [19]. From this perspective, tubules with thin glycocalyces may be more susceptible to injury than glomerular vessels with thicker glycocalyces.

### Renal capillary glycocalyx injury in rhabdomyolysis-induced AKI

The luminal surface of healthy vascular endothelial cells contains a glycocalyx composed of polysaccharides and glycoproteins [20, 21]. The glycocalyx is composed of proteoglycans, glycosaminoglycans, and sialoproteins that bind to vascular endothelial cells and play important roles in regulating blood flow, including the regulation of microvascular tone and permeability, leukocyte adhesion and migration, and intravascular thrombi.

However, when the glycocalyx of the vascular endothelium is disrupted by inflammation, vascular permeability increases, and vascular contents leak out of the vessels [19]. In addition, platelets and inflammatory cells adhere to vascular endothelial cells, disrupting blood flow and the extravasation of inflammatory cells. In rhabdomyolysis-induced AKI, the endothelial glycocalyx of the capillaries may be injured, which causes inflammation between the capillaries and tubules, resulting in voids and fibrosis. Moreover, the impairment of the vascular endothelial glycocalyx can cause perivascular voids [22]. Increased vascular permeability may be the cause of voids between the capillaries and tubules.

### Tubular protective effects of recombinant antithrombin

AT is not only an inhibitor of serine proteases in the coagulation cascade, such as thrombin, but also has anti-inflammatory and cytoprotective effects [23–25]. AT decreases renal tumor necrosis factor-alpha, monocyte chemoattractant protein-1, and intercellular adhesion molecule-1 expression, which is implicated in local inflammation [26], suggesting that AT is an important anti-inflammatory mediator of renal inflammation. The mechanisms of AT anti-inflammatory effects include the inhibition of neutrophil recruitment and leukocyte rolling and infiltration [27, 28], the inhibition of cytokine secretion [29], and the protection of the vascular endothelium [30]. In the present study, a decrease in inflammatory cell infiltration was observed in the rAT-treated group, confirming the anti-inflammatory effects of rAT.

Furthermore, in this study, tubular regeneration was observed in the tubules of the mice in the rAT-treated group. In addition, in our recent study, we reported that rAT exerts DNA-repair effects [10]. In this study, many PCNA- and Ki-67-positive cells involved in the cell cycle were observed in the rAT-treated group.

### Limitations

A limitation of the current study is that tubular regeneration may have occurred due to the tubule repair/regeneration-promoting action of rAT itself or due to the inhibition of glycosylation damage in the vascular endothelium, maintaining microcirculation and causing tissue repair. Therefore, further detailed studies are required to verify the results and claims of our study. Likewise, the present study was conducted only in male mice first to confirm the effects of rAT on the kidneys, excluding effects by sex (since female hormones are known to affect vascular endothelial glycocalyx), and more detailed studies should be conducted in the future to demonstrate differences by sex. In addition, serum and urine testing is needed to quantitatively assess the shedding of glycocalyx, including syndecan-1 in the future. Electron microscopy is limited to observation of a very small area; new methods for quantification of microvilli should be considered in the future.

## Conclusion

Treatment with rAT attenuates proximal tubular injury through several factors, including anti-inflammation and accelerated restoration of endothelial cells and proximal tubules. Since rAT is already used in clinical applications, these results could lead to a new strategy for treating AKI due to rhabdomyolysis.

### Supplementary Information

**Additional file 1: a. **Representative images of the immunohistochemical staining for PCNA in the kidneys of sham-operated mice and mice treated with saline or rAT. **b.** Graph showing the number of PCNA-positive cells (n = 6 in each group). *P < 0.05 vs. Sham-operated mice. + *P* < 0.05 vs. Saline-treated mice.

**Additional file 2. **Representative images of the brush border using a transmission electron microscope for sham-operated mice (**panel a**), mice treated with saline (**panel b**), rAT (**panel c**). **d:** Graph showing the length of the villus of the brush border (*n* = 6 in each group). *P < 0.05 vs. sham-operated mice. + *P* < 0.05 vs. saline-treated mice.

**Additional file 3: **The graph of the intensity of WGA lectin staining in CD31-positive areas in the capillaries around the tubules. *P < 0.01.

## Data Availability

The data sets supporting the conclusions of this article are included within the article (and its additional files).

## Abbreviations

AKI: Acute kidney injury
AT: Antithrombin III
N-GAL: Neutrophil gelatinase-associated lipocalin
rAT: Recombinant antithrombin
