# Supplementary figures and images for: Recombinant antithrombin attenuates acute kidney injury associated with rhabdomyolysis: an in vivo animal study
Source: Intensive Care Med Exp. 2024 Jan 29;12:7. doi: 10.1186/s40635-024-00594-y (PMC10822833; doi:10.1186/s40635-024-00594-y)

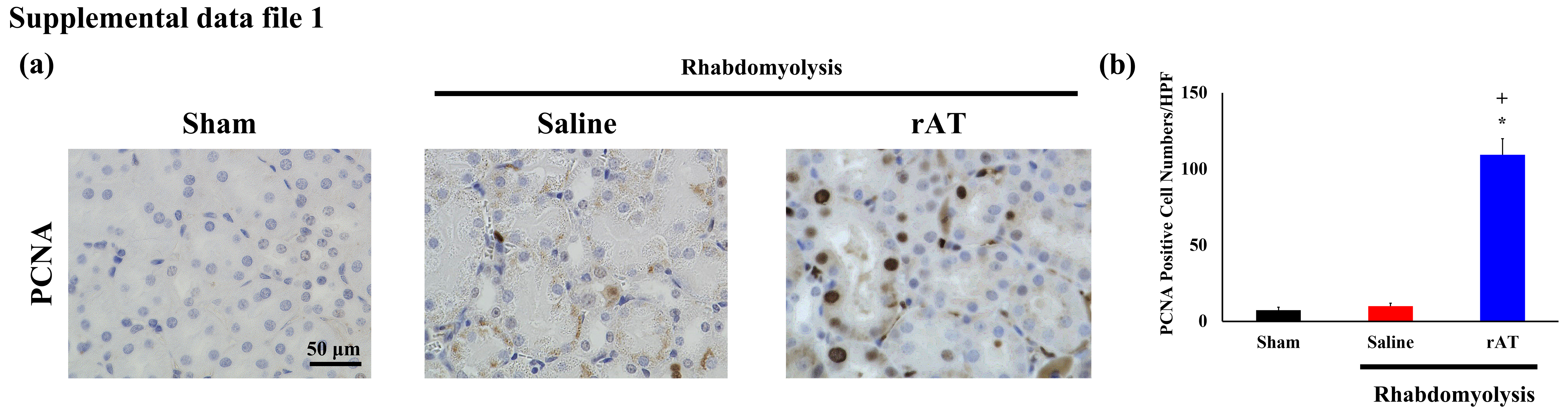

Supplement: Supplementary file 1 — Additional file 1: a. Representative images of the immunohistochemical staining for PCNA in the kidneys of sham-operated mice and mice treated with saline or rAT. b. Graph showing the number of PCNA-positive cells (n = 6 in each group). *P < 0.05 vs. Sham-operated mice. + P < 0.05 vs. Saline-treated mice. [file 40635_2024_594_MOESM1_ESM.tif]

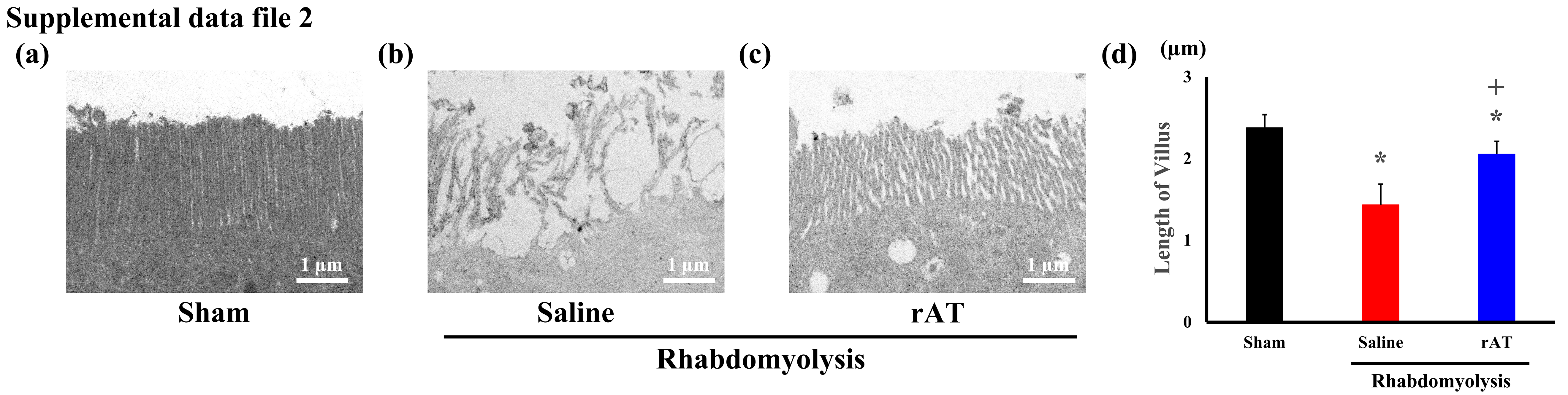

Supplement: Supplementary file 2 — Additional file 2. Representative images of the brush border using a transmission electron microscope for sham-operated mice (panel a), mice treated with saline (panel b), rAT (panel c). d: Graph showing the length of the villus of the brush border (n = 6 in each group). *P < 0.05 vs. sham-operated mice. + P < 0.05 vs. saline-treated mice. [file 40635_2024_594_MOESM2_ESM.tif]

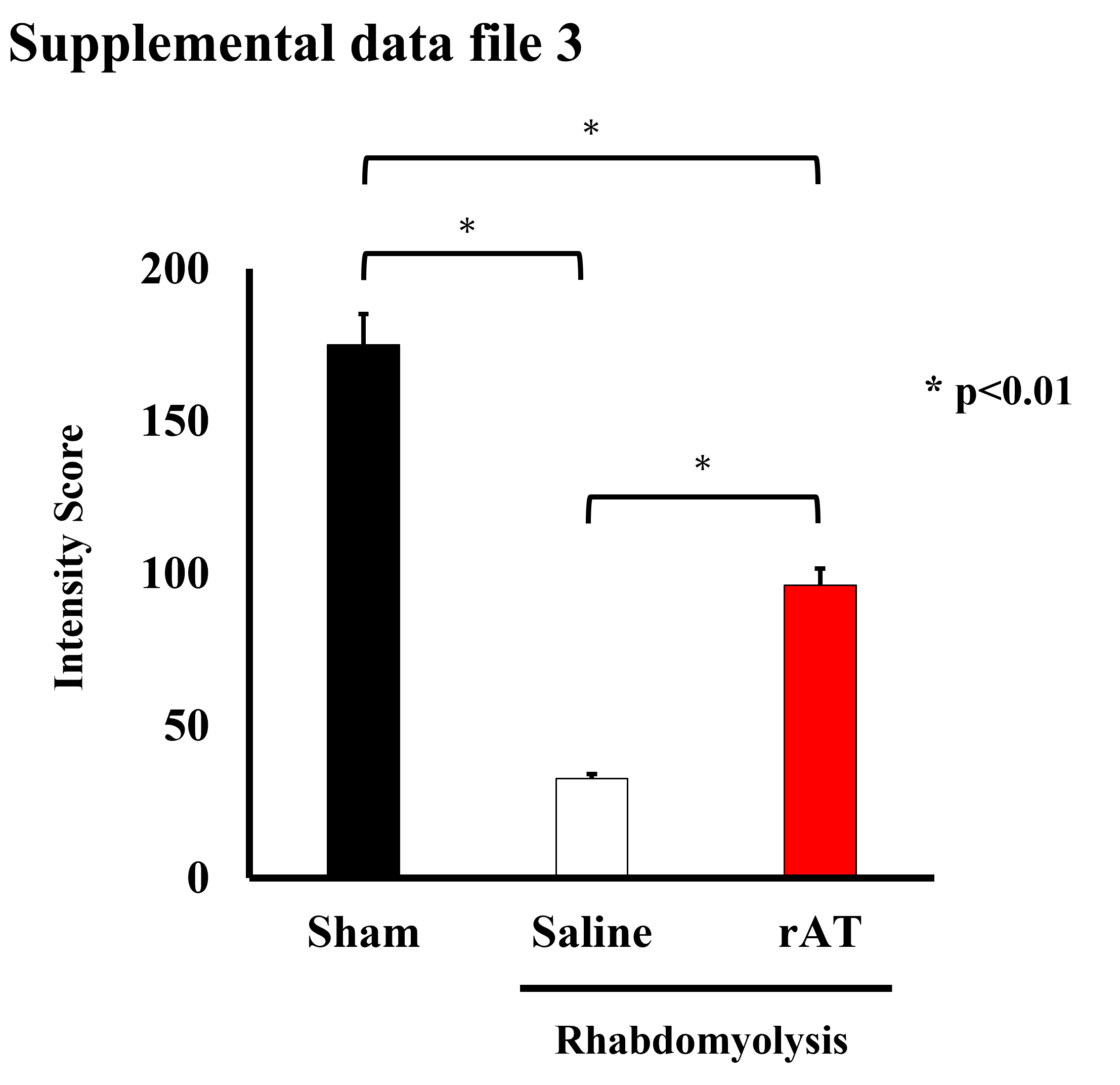

Supplement: Supplementary file 3 — Additional file 3: The graph of the intensity of WGA lectin staining in CD31-positive areas in the capillaries around the tubules. *P < 0.01. [file 40635_2024_594_MOESM3_ESM.tif]
